# Peer review of "Reanalyzing Head et al. (2015): investigating the robustness of widespread p-hacking"

_PeerJ, doi:10.7717/peerj.3068_

## Round 0.1 · original submission · Major Revisions

Three reviewers have highlighted several major issues in regards to the analysis and presentation of your manuscript. I have carefully read their reviews, your manuscript, and Head et al, and believe that major revisions are in order.

Reviewer 1 makes a valid point about the possible lack of power of your test (relative to Head et al), which should be discussed. Reviewer 2 feels that more work needs to be done to determine the robustness of Head et al's results. Reviewer 3 is generally positive about your manuscript.

It seems to me that much of the main disagreement between your work and Head et al is the proper way to bin data. Since that is the case, I think that summarizing multiple binning strategies or using a test of uniformity that doesn't requiring binning (e.g. Kolmogorov-Smirnov) would satisfy the reviewers and improve the analysis.

Please respond to all the points of the reviewers in a detailed rebuttal and revised document.

·

Basic reporting

See my PDF review for all comments.

Experimental design

See my PDF review for all comments.

Validity of the findings

See my PDF review for all comments.

Additional comments

See my PDF review for all comments.

·

Basic reporting

This is a paper on an important topic. A large number of papers on p-curve analyses have appeared over the last couple of years. The work by Head et al. stands out, having already accrued 62 citations in Google Scholar (24 September 2016). It is fair to have a critical look at their methods and conclusions.

However, I find this commentary not fruitful in present form. I have three main comments.

1. As far as I understand, the crucial difference between your approach and that of Head et al. is that Head et al. compared p-values 0.041, 0.042, 0.043, 0.044 versus 0.046, 0.047, 0.048, and 0.049 (I assume that p-values with four or more digits are very rare). The approach of Head et al. seems reasonable to me, because 4 p-values of assumed equal reporting style are lumped and compared with each other (although one should wonder why they excluded 0.045, and not e.g., 0.041).
In your analysis you effectively compare p = 0.04 with p = 0.05 (combined with 3-digit p-values just below 0.04 and just below 0.05, respectively. These 3-digit p-values do not change the overall picture; the largest share of the effect can be attributed to the 2-digit p-values, see Table 1). Comparing 0.04 with 0.05 seems a dangerous activity, because 0.05 is on the verge of statistical significance, and may therefore be susceptible to issues of variable reporting styles such as p = 0.05, p < 0.05, p > 0.05). This can also be seen in your Figure 1, where 0.05 is considerably rarer than 0.04. There seems to be no explanation for this drop from 0.04 to 0.05. It seems plausible to me that if researchers find p = 0.052 they may (and should) report p > 0.05.
You do not seem to justify clearly why your approach is preferred over the one of Head et al., nor do you explain what could cause the difference (e.g., why is p = 0.05 relatively rare?). In the limitations section of this work it is pointed out that ‘rounding’ (e.g., researchers reporting p < 0.05 instead of p = 0.047) could be a cause. I agree, but isn't this a very severe limitation?
So, although I can agree that the results of Head et al. are dependent on how you look at their data, there appear to be no indications that they did anything wrong or even suboptimal.
2. Related to the previous point, this commentary does not offer much of a ‘big picture’. I find the work generally light on citations and interpretations. For example, the target article of Head et al. (2015) has been (indirectly) criticized before
Bruns, S. B., & Ioannidis, J. P. (2016). p-curve and p-hacking in observational research. PLOS ONE, 11, e0149144.
Furthermore, the work of Head et al. has been discussed online.
https://peerj.com/preprints/1266v1/#feedback
I believe that if a commentary is submitted, then it should also review previous commentaries/discussions on the same paper and present some more context.
Also, because you are concerned with issues of ‘robustness’, it seems opportune to have a brief look at other papers that were concerned with the ‘bump’. Does your work imply that other studies that use p-curve analyses cannot be trusted either? There is a certain school of thought that argues that looking at aggregate p-curves is a meaningless activity, but you do not seem to belong to this school of thought by stating “a bump below .05 is a sufficient condition for the presence of specific forms of p-hacking.” . Then again, you appear to contradict yourself by concluding: “if any detection of p-hacking is attempted, this should be done at the paper level and after careful scrutiny of which results are included (Simonsohn et al., 2015; Bishop and Thompson, 2016).” What are the implications of this work?
3. Third, this manuscript is hard to follow. For example, it is not so clear which strings were included in your analysis. If you say e.g., “.003875 < p <= .04”, does this include “p = 0.04” as well as “p < 0.04” (I assume not) as well as “p = 0.040”? Later on you say “t(59) = 1.75, p > .05 was included”. Did you really include the ‘greater than’ symbol in your analyses or does this refer to the work of Head et al. only? Only in the conclusion/recommendations section, it says that yours were a “selection of only exactly reported p-values” Elsewhere in the paper (Table 1) you use a symmetric notation “(.03−.04)” while .04 was included and .03 was not.
Similarly, I had a very hard time interpreting Fig. 1.
a. The ticks do not seem to be aligned properly. I assume that the tall bars around 0.01, 0.02, 0.03, 0.04, and 0.05 are due to people reporting p-values up to two decimals, but the tick markers are right next to these bars, rather than below it.
b. The figure caption says “(in grey; bandwidth = 0.00125).” Similarly, in the text it says “Visually, the newly selected data the grey and black bins from Figure 1 combined, where the rightmost black bin (i.e., .04875 < p ≤ .05) is compared with the large grey bin at .04 (i.e., .03875 < p ≤ .04).” However, I count 50 grey bars, and 50 * 0.00125 = 0.0625, not 0.05. Perhaps the ‘tall bars’ refer to p-values with two decimal places, and the bars in between represent bins of 0.00125 wide? If this is the case, then I count 45 bars, which still does not add up to 0.05.
c. The text says: “The two green bins (i.e., the sum of the grey bins in the same range) show a bump below .05, which indicates p-hacking”. In the figure, the green bars span the width also of the black bars, making this very confusing. Furthermore, in histograms the area counts (https://en.wikipedia.org/wiki/Histogram), but here we should not be looking at the area of the green bars, but at the height only.
d. Did the authors distinguish between e.g., p = 0.04 and p = 0.040? or are these visible in the same bar? As mentioned above, it is not explicitly clear how e.g. < was handled, or >. E.g., is the first bin p < 0.001, p = 0.001, or p = 0.000.
e. I am quite unclear what the difference is between the black bars and the grey bars. The black bars appear to be stacked on top of the (tiny) grey bars (i.e., a tiny grey bar can still be seen). I assume this is just a plotting issue, and not supposed to be like this.
f. All in all, this figure is inappropriate in its current form. For each bar, it should be clear what it actually means (which p-value strings/range it represents). Without clear information about what is actually reported in the figure, the whole paper becomes rather difficult to interpret.
A few minor issues (not comprehensive)
• Lines 11-12. This appears to be grammatically ambiguous. More than what?
• Line 13. Again, more than what?
• Line 16. It may not be clear for a reader what you mean here: “when we take into account a second-decimal reporting tendency”
• Lines 17-20. These sentences can be improved, and appear to be grammatically incorrect/convoluted (e.g., “given that … it is important that … if the conclusion is…”
• Line 30. Could you specify what Simonsohn et al. stated? What “epistemological concerns”?
• Line 36. Inappropriate comma
• Line 37. Equating left skew with a bump is not precise. Left skew (i.e., negative skew) has a formal definition. Also, it may be useful to define such bump in formal terms (which range are we talking about and does the bump always have to be approached from the left?
• Line 41. Why “consequently” ?
• Line 42. Stating that a bump below 0.05 is a sufficient condition for the presence of p-hacking is a strong statement. Here, you may be more precise. For example, there may be sampling error (chance effects) or specific types of study heterogeneity that may invalidate such claim (see also Bruns & Ioannidis, 2016). Also. it should be made clear whether you refer to a theoretical assumption or to an observation of p-values.
• Line 65. There is a grammar error in this sentence.
• Line 77. The word ‘significant’ may be somewhat inappropriate if not specifying the alpha value. That is, the implicit assumption is that everybody used alpha = 0.05, but this may not be the case.
• Line 78. At this point in the text you have not explained what are “those results depicted in black”
• The keywords ‘nhst’ and ‘grps’ (questionable research practices?) are a bit peculiar.

Experimental design

-

Validity of the findings

-

Additional comments

-

·

Basic reporting

Well written

Experimental design

Not relevant

Validity of the findings

Justifiable decisions in the analytic strategy

Additional comments

This short manuscript makes a single point: When the data by Head et al (2015) is reanalyzed based on extremely justifiable decisions, such as including p = 0.05 and taking into account that p-values are more likely to be reported as 2 digits, there is no evidence for widespread p-hacking. Given the response to the original article, and the citations it has been getting, this seems like a useful fact to point out to the scientific community.

I only have one question for clarification. The Bayesian Calipher test is not clearly explained in lines 118 and beyond, so I don’t understand why I should be interested in these results. I don’t understand what the prior is, or why a simple likelihood is not a plausible alternative. More information is needed for readers to be able to understand this test.

It might be worth pointing out that there are now many additional datasets, or re-analyses, that do not reveal the ‘bump’. We don’t have a way to meta-analyze such datasets, butt to me, it seems as if more papers did not find the bump, than that there were papers that found the bump. This should give additional weight to the current observation that the bump disappears based on justifiable choices for a different analysis strategy.


Wording:
Line 42: Questionable behaviors seeking just statistically significant results,

---

## Round 0.2 · Major Revisions

I have reviewed the revisions and the comments made by the three reviewers. The reviewers have divergent opinions about the manuscript, and the paper needs further revisions to satisfy reviewers 1 and 2, especially issues surrounding the treatment of p=0.05, and the interpretation of the results.

As reviewer 2 points out, Head et al. concluded that the effect of p-value hacking was weak, so it not surprising to me that alternative study designs, with potentially less power, may not produce a significant result. This needs to be addressed further in your discussion. If possible, using robust resampling/simulations to compare the power of your binning strategies vs Head et al. would help resolve the differences between you and reviewer 1.

Please respond to all the points of the reviewers in a detailed rebuttal and revised document.

·

Basic reporting

See my PDF review

Experimental design

See my PDF review

Validity of the findings

See my PDF review

Additional comments

See my PDF review

·

Basic reporting

The manuscript has undergone several improvements, especially when it comes to more precise reporting of numbers and formatting of the figure. Still, the caption of Figure 1 can be expanded to make even clearer what can be seen. For example, a reader now has to infer that p = 0.005 belongs to the fourth bin (I presume), not the fifth (e.g., try to explicitly use < vs. ≤ throughout; e.g., in Table 1 no such symbols are used). There is a related issue: In the previous manuscript, the p-curve looked smooth, while in the revised manuscript there are distinct bumps at 0.005, 0.015, 0.025, 0.035, and 0.045. This is most likely a result of the altered binning process (e.g., 0.004 and 0.005 being part of the same bin?). This issue is quite confusing, especially because the inclusion/exclusion of 0.045 is a central theme of your work. Please clarify.

Second, it is unclear to me how the Fisher test was applied. It is stated that “all p-values between .04-0.05” were submitted to the test, which is unclear to me. What are ‘all p-values’?

Furthermore, I have difficulty with another aspect of this paper. While I believe that the manuscript provides an informative read (especially when reading it in combination with the comments/simulations of Reviewer 1 & Rebuttal), I do not understand your treatment of p = 0.05.

Looking at our own work (De Winter & Dodou, 2015, Fig. 22), we found the following frequencies of p-value strings in abstracts:

p = 0.03: 7,047 times
p = 0.04: 6,211 times
p = 0.05: 3,162 times
p < 0.05: 40,904 times
p > 0.05: 7,255 times

In other words, it appears to me that the alpha = 0.05 threshold has a large influence on how people report p-values. It is unclear to me why you present this as a discussion point rather than as an integral part of your work. In the discussion you state “There is SOME indication that this rounding tendency is a BIT stronger around .05 than around .04” (emphasis added) while at the same time referring to a 25% difference.

To sum up, this paper contains interesting information, but there are aspects that can be improved, especially a reconsideration/motivation regarding whether p = 0.05 should be included in your comparison. My interpretation of your Figure 1 is that Head et al. found some evidence of a bump, but that the effect is weak compared to the effects of reporting style (e.g., whether one reports p < .05, p = 0.05, or p > 0.05). So while I agree that a small effect found by Head et al. may not be robust for several reasons, I am not sure whether including p = 0.05 is a good idea.

BTW, interestingly, looking at the abstract of Head et al., they in fact reach a similar conclusion “We then illustrate how one can test for p-hacking when performing a meta-analysis and show that, while p-hacking is probably common, its effect seems to be WEAK relative to the real effect sizes being measured” (emphasis added)

Experimental design

-

Validity of the findings

-

Additional comments

-

·

Basic reporting

OK

Experimental design

NA

Validity of the findings

This re-analysis reveals important additional considerations when evaluating Head et al. The analytic choices seems valid to me.

Additional comments

I only had minor comments to the original manuscript, and I have no further comments on this version – I think it is suited for publication.

I’ve also through the rebuttal letter’s comments addressing the other reviewers concerns. I find the arguments of reviewer 1 that people round differently near 0.05 unlikely – as far as I know there is no empirical data on this. There seems to be a lively debate about how many digits should be used for p-values – my recommendation is to always use 3. I have never heard anyone recommend to use 2, except when you are near 0.05. The second argument (that people do not round down p = 0.054) is incorrect - there is an application called StatCheck that Hartgerink is a co-author on that shows this happens a lot.

In the end, the contribution of this commentary article is straightforward, and since there are now more papers showing there is no ‘bump’ just below 0.05 after correct analyses I think it is important this analysis appears in press. I’m slightly dismayed by the tenacity of the original author to prevent other viewpoints than his own from entering the literature. The argument here is very straightforward, there is no reason why the analytic strategy in the original article is more defensible than the choices by Hartgerink (if anything, the reverse seems true to me) and I personally feel competent enough to compare both approaches and decide which I find more reasonable – the scientific community should have access to this information.

---

## Round 0.3 · accepted · Accept

Both reviewers have indicated that you have cleared up most issues with your manuscript. Reviewer 2 has indicated that you need to make two sections a bit clearer, and that can be handled in the production stage.

Thank you for providing the equation for "Fisher's Test". I now see that you are testing the null that the p-values between 0.40 and 0.50 are distributed uniformly, with the alternative being that there are more p-values towards 0.50. I was confused about your method because I read "Fisher's Test" as "Fisher's Exact Test". You can use "Fisher's Method" to reduce the confusion. (https://en.wikipedia.org/wiki/Fisher's_method). I also found https://dx.doi.org/10.2307%2F2681650 to be a valid citation for the method.

I am comfortable that the power of your analysis is not significantly weaker than what Head et al. used. A quick-and-dirty estimate of how strong the signal of p-hacking must be to detect an effect suggests that Fisher's method is more sensitive if the p-hacking increases the number of values above 0.046. When it is less sensitive, it can still detect signals of roughly the same magnitude as the binomial test. Another advantage of Fisher's method is that you don't have to worry about binning strategies and values that fall on the edge of bins, due to rounding. See below for the code that I used to compare the methods, which could be wrong/buggy.

```
# model: data is a mixture of two uniform distributions
# component one has probability (x-b) and is distributed in [0,x)
# component two has probability (1-x+b) and is distributed in [x,1)
#
# goal: Find how big b needs to be to expect a significant result based on x and sample size
#

len = 38246
xx = seq(0.01,0.99,0.01)

# Test 1: Bin data in [0,0.5) and [0.5,1) and test if there is an excess in the upper bin
#
binsig = qbinom(0.95,len,0.5)
biasBinom = c()
for(x in xx) {
result = uniroot(function(b) { len*ifelse(x < 0.5, (1+b-x)/(2-2*x), (b+x)/(2*x))-binsig},c(x-1,x))
biasBinom = c(biasBinom,result$root)

}

# Test 2: Fisher's Method to see if there are excess upper values
#
f = function(a,b) {
aa = ifelse(a < 1, (1-a)*log(1-a), 0)
bb = ifelse(b < 1, (1-b)*log(1-b), 0)
(2*(a - b + aa - bb))/(a - b)
}

x2sig = qchisq(0.95,2*len) # 77139.49
biasFish = c()
for(x in xx) {
result = uniroot(function(b) { len*((x-b)*f(0,x)+(1-x+b)*f(x,1))-x2sig},c(x-1,x))
biasFish = c(biasFish,result$root)

}

matplot(cbind(biasFish,biasBinom))
```

·

Basic reporting

I am inclined to accept this article provided the last 2 comments are cleared up (scroll down). I agree with the statement made by the author “I am afraid that further discussion will obfuscate or polarize the review process further.”

Lots of words have been written by the reviewers and in rebuttal letters, but few answers have been provided (perhaps because the complexity of this topic does not allow for answers). The author sometimes appears to take an easy way out by making only minor changes to the manuscript. For example, although Reviewers 1 and 2 have questions about the potential quantitative impact of rounding of p values, and various literatures is invoked, the actual change made to the manuscript is merely a change of the words ‘rounding tendency is a bit stronger’ into ‘tendency to round up is relatively stronger’. However, I believe that Reviewer 1 (the author of the targeted article) is not particularly clear either, by invoking a large number of arguments and simulations which appear to distract from the overall message.

What I find disappointing is that we are left with a number of descriptive tables/figure, and no in-depth insight. It should have been possible to come up with more answers, like why Head et al. found ‘a bump’ and other crawling papers did not? On a positive note, I am happy that some technical errors have been cleared up. We are now left with a manuscript that appears to be technically correct (I also like the fact that source code is available), but subject to disparate interpretations (which is fine).

What would perhaps be useful for the record (what it is worth) is to give my interpretation of ‘the bump’ in simple words.
* Head et al. claimed to have identified ‘the bump’.
* As correctly pointed out by Reviewer 3, other papers did not find such bump (including myself). Then again Krawczyk (2015) did identify QRPs based on p values.
* If manually inspecting p-values in the literature, one may notice that lots of factors could explain a bump, such as wrongly calculated p-values, interactions with publication bias, different reporting styles (e.g., a tendency to write out p-values to 3 digits if they are near 0.05, and even nuisance p values (e.g., in physical oriented papers, P might refer to power). To make things worse, statistical power and reporting styles (and corresponding effect size measures) differ between research fields. Because effect sizes in the 0.04 to 0.05 region are small compared to other effects (e.g., the major drop from p < .05 to p >.05), I agree that the bump is a delicate and perhaps even entirely spurious finding. I have engaged in such discussions before as well with colleagues (e.g., can you infer p-hacking from aggregate collections of p-values?), and I have learned that such discussions usually turn out indecisive (like the present commentary vs. Reviewer 1) and highly dependent on what you would consider evidence. For a good read, see Ioannidis JP. 2014. Discussion: why “An estimate of the science-wise false discovery rate and application to the top medical literature” is false. Biostatistics 15:28–36. dx.doi.org/10.1093/biostatistics/kxt036
* The main analysis of Hartgerink is a simple one. He did not exclude .045 or .050. What is a bit ungratifying is that there is no explanation for this decision. Is it good or bad? What we are left with is the following: “I am not trying to say that the Head paper is correct or incorrect or that my approach is better, but simply that it is not as unequivocal as it was presented. It is up to the readers to decide what they find more convincing.” Although this is technically a correct statement (how could it be incorrect?), it is also a lukewarm one. It is a bit like a doctor saying to a patient: “you may not be ill after all” because my non-validated measurement tool did not detect a disease. I think a reductionist approach (i.e., go back to the individual papers, see what is going on) could potentially resolve the dilemma in future research.

Some remaining comments.
* I still do not find it clear how the Fisher test was used. Reference is provided to a book from 1925 where the equation you report could not be found. Was not clear to me what is actually being tested here, and what the statistical power may be for said test, and what k and df are. The corresponding sentence is grammatically incorrect “The Fisher test was … and tested for …” “are … are transformed”.
* What exactly do you mean by “Taking into account reporting tendencies is important…”

Experimental design

-

Validity of the findings

-

Additional comments

-

·

Basic reporting

OK

Experimental design

Not relevant

Validity of the findings

Valid, since the main point it that changes to the assumptions matter.

Additional comments

I have read the rebuttal. I have also been contacted by Reviewer 1 through email, who has explained his viewpoint to me. Here, in what will be my final review of this paper, which has taken up more time than I think is warranted, I want to explain one final time why I think this commentary is ready for publication.

My most important reason for this is the fact that there is no indication of a ‘bump’ in many other datasets in the literature. Another reference that is missing from the current paper is:
Vermeulen IE, Beukeboom CJ, Batenburg AE, Stoyanov D, Avramiea A, Van de Velde RN,
Oegema D. 2015. Blinded by the light: p-value misreporting and excess p-values just below
.05 in communication science.
These authors also did not find a ‘bump’. These authors also show rounding down for e.g., p = 0.053 happens, which is worthwhile to cite. The paper by Krawczyk was also new to me. Note that the paper by Krawczyk also does not show a ‘bump’ (Figure 3). Indeed, the difference between Figure 2 (based on reported p-values) and Figure 3 (based on recalculated p-values) in Krawczyk to me pretty much confirms that there is no bump, but that there is a rounding issue. Figure 2 replicated the findings by Head et al (we see more reported p-values in the 0.046-0.049 bins than the 0.041-0.044 bins. This completely disappears when we look at the recalculated p-values in Figure 3 based on the text statistics from these papers. If people were p-hacking, the bump should be in the p-values AND the test statistics. If this is a rounding issue, it should only be visible in the reported p-values. Since we only see it in the reported p-values, I think it is safe to conclude rounding matters, and the main point that Hartgerink makes that different ways of dealing with rounding changes the results is worthwhile to share.

In his email to me, Reviewer 1 remarked: “you and me seem to agree that there is decent empirical evidence people round off in a biased way near p = 0.05”. Yes, but I think we know so very little about people’s rounding behavior over the entire p-value range, and so much hinges on the ‘ifs and mights’ about this behavior raised by Reviewer 1, and posited in Head et al, that I think this commentary that changes the ‘ifs and mights’ in a reasonable way should be published. This will 1) communicate to researchers that the original claims in Head et al depend on assumptions that are debatable, and that the effect is not present under different reasonable assumptions, and 2) make it clear why drawing conclusions about p-values is so difficult. This might motivate researchers who really want to do draw conclusions about such large sets of p-values to study p-value rounding behavior in more detail, and collect real data, instead of having the prolonged and impossible to resolve discussions as we have seen here.

In this dataset there is a remarkable, and unexplained, large (compared to expectations) difference between the p = 0.04 and p = 0.05 bins. This difference was not noticeable in Head et al, and I think it is worth publishing this commentary only for Figure 1. We can discuss how and why people round p-values for a long time, or we can publish this commentary, and motivate some researchers to collect empirical data on this question. The statement in Head et al that “Here, we use text-mining to demonstrate that p-hacking is widespread throughout science” seems too strong to me, and needs to be nuanced, and this commentary serves that goal. The ‘mights’ and ‘ifs’ by reviewer 1 and the author are a truly academic discussion as long as there is no data, and I’m not surprised the reviewer and author can not reach agreement – there is not enough data to reach agreement, which also means there is no hard enough data to make strong claims about this dataset. So we have Head et al arguing p-hacking is widespread, and Hartgerink arguing that if the data is analyzed in a different (reasonable) way, there is no evidence of widespread p-hacking, and both could be true, depending on how people actually round p-values, which we don’t know.

Another clear indication that people have weird rounding behavior is raised by Reviewer 2, and it is the increased frequency of p-values around 0.0X5 (e.g., 0.045, 0.035, etc). The author can not explain this. Head et al do not explain this, but exclude p = 0.045 without a clear justification. We probably need data to explain this. But let me make 1 wild guess. People like reasonably round numbers. They don’t have strong reporting default (e.g., ‘always report p-values to 3 digits’). So they round ‘intuitively’. They like to round to two digits (clear in the data), but clearly also to 3 digits, especially to 0.0X5 (clear in the data). BUT: they have an overall small preference to round to 3 digits in the range of 0.046 to 0.049 just to clearly communicate the p-value is really below 0.05. The assumption made in Head et al (2015) is that “we suspect that a p-value is more likely to be abbreviated to p < 0.05 if it is p = 0.049 rather than, say, p = 0.041” There is no evidence for this. I think people are more likely to report p = 0.049 than p = 0.05. If we combine the idea that people slightly prefer reporting exact p-values from 0.046 to 0.049, with the clearly visible pattern that they also like to round to p = 0.045, the entire ‘bump’ is explained away by how people round p-values. Again, this idea is supported by the lack of a bump in the recalculated p-values in Krawczyk.
Therefore, I think a commentary that makes the point that “Moreover, the elimination of p = .045 and p = .05, as done in the original paper, is debatable.” and: “These results nuance the conclusion by Head et al. (2015), indicating that the results are not robust and that the evidence for widespread left-skew p-hacking is ambiguous at best.” deserves a place in the literature.